# Optimization of modified CT severity index by incorporating lymphocyte-to-monocyte ratio for early stratification in acute pancreatitis: a single-center validation study

**DOI:** 10.3389/fmed.2026.1752594

**Published:** 2026-01-26

**Authors:** Li Zhang, Xiao Cao, Baochun Wang, Peng Ji

**Affiliations:** Hefei Third People’s Hospital, Hefei, China

**Keywords:** acute pancreatitis, early stratification, lymphocyte-to-monocyte ratio, modified CT severity index, predictive model

## Abstract

**Background:**

Acute pancreatitis is a common digestive system emergency with heterogeneous clinical courses, and early accurate stratification is crucial for guiding treatment, improving prognosis, and reducing mortality. Existing scoring systems such as the Modified CT Severity Index (MCTSI) primarily rely on imaging features, but alone may not fully reflect inflammatory status, while inflammatory markers like the Lymphocyte-to-Monocyte Ratio (LMR) show potential in predicting disease severity; however, studies combining both are insufficient.

**Objective:**

To investigate the value of integrating LMR with MCTSI to construct a new scoring system (MCTSI-LMR) for early stratification in acute pancreatitis, optimizing clinical predictive efficacy.

**Methods:**

A total of 216 patients with acute pancreatitis admitted to Hefei Third People’s Hospital from April 2022 to January 2025 were selected as the study subjects. All patients were divided into mild group (65 cases), moderately severe group (108 cases), and severe group (43 cases) according to the Atlanta classification criteria (2012 revision). All patients underwent abdominal CT scanning and complete blood count within 24 hours of admission to calculate MCTSI scores and LMR values. Using clinical outcomes as the gold standard, inter-group indicator differences and the diagnostic efficacy of the combined score were compared.

**Results:**

Based on disease severity, the severe group had significantly lower LMR values than the mild and moderately severe groups (*t* = 125.473, *P* < 0.001) and higher MCTSI scores (*t* = 298.456, *P* < 0.001). The incidence of organ failure and mortality showed statistically significant differences between groups (χ^2^ = 98.765, *P* < 0.001; χ^2^ = 45.678, *P* < 0.001). Multivariate logistic regression analysis revealed that MCTSI score (Wald = 26.234, *P* < 0.001) and LMR (Wald = 41.156, *P* < 0.001) were independent predictors of severe acute pancreatitis. ROC curve analysis indicated that the MCTSI-LMR score had superior predictive performance compared to individual indicators (*Z* = 3.456–5.678, *P* < 0.05).

**Conclusion:**

The application of the MCTSI-LMR score in early stratification of acute pancreatitis can significantly enhance predictive efficacy, with notable differences combining imaging and inflammatory markers, providing a reliable tool for clinical early intervention and worthy of promotion and application.

## 1 Introduction

Acute pancreatitis (AP) is a common clinical abdominal emergency characterized by rapid progression and significant heterogeneity. Early and accurate stratification is critical for guiding treatment and improving prognosis (1). The modified computed tomography severity index (MCTSI), widely used in clinical practice, grades disease severity by assessing morphological changes in pancreatic and peripancreatic tissues (e.g., pancreatic necrosis, fluid collections), offering high objectivity and reproducibility (2). However, MCTSI relies primarily on imaging features and fails to adequately incorporate biomarkers reflecting systemic inflammatory responses, potentially limiting its sensitivity and comprehensiveness in evaluating early inflammatory states (3).

In recent years, hematological inflammatory markers have garnered attention due to their accessibility and ability to dynamically reflect immune status. The lymphocyte-to-monocyte ratio (LMR), a potential indicator of systemic inflammation, has demonstrated prognostic value in various inflammatory diseases (4). Studies suggest that decreased LMR is closely associated with excessive systemic inflammation and immune dysregulation, possibly indicating an increased risk of organ failure in AP patients (5).

Although MCTSI is widely applied in AP severity assessment, its sole reliance on imaging parameters restricts its capacity to capture early inflammatory bursts and dynamic immune responses (6). Conversely, while LMR reflects immune-inflammatory status, as a standalone hematological parameter, it is susceptible to interference from non-specific factors (e.g., concurrent infections, underlying hematological disorders) and lacks integration with imaging findings (7). Most existing studies have focused solely on either imaging or hematological indices, overlooking the potential of multi-dimensional data integration (8). Furthermore, current combined models are predominantly based on retrospective data and lack rigorous prospective validation, raising concerns regarding their clinical applicability and robustness (9). Notably, inflammatory responses and local tissue injury interact spatiotemporally during AP progression; assessment from a single dimension may underestimate the true disease risk (10). Therefore, developing an integrated tool combining morphological and immunological indicators may address the limitations of existing stratification systems and enhance early warning efficacy (11).

This study aims to develop a novel combined scoring system, MCTSI-LMR, by integrating MCTSI and LMR, and validate its efficacy for early stratification of AP in a single-center clinical cohort. We hypothesize that MCTSI-LMR synergistically reflects structural damage and systemic inflammatory status, thereby providing a more comprehensive and dynamic risk assessment. By comparing the predictive performance of the combined score against conventional indicators for organ failure and clinical outcomes, this study seeks to provide new evidence for early precision stratification in AP and promote the integration of multi-modal data in clinical decision-making.

## 2 Materials and methods

### 2.1 General Information

This single-center, prospective cohort study was conducted at Hefei Third People’s Hospital, Anhui Province, from April 2022 to January 2025, and enrolled 216 patients with acute pancreatitis. The cohort included 147 males and 69 females, with ages ranging from 19 to 93 years (mean age 46.55 ± 16.11 years). Sample size estimation was performed using G*Power software (version 3.1) for *a priori* power analysis. Parameters were set as follows: significance level α = 0.05, power β = 0.20 (80% power), and effect size *d* = 0.5 (medium effect), based on preliminary experiments. The minimum required sample size was calculated as 200 patients; ultimately, 216 patients were included to account for potential dropouts or missing data, ensuring statistical robustness.

Patient stratification followed the revised Atlanta classification (2012), resulting in 65 cases (30%) with mild acute pancreatitis, 108 cases (50%) with moderately severe acute pancreatitis, and 43 cases (20%) with severe acute pancreatitis. Demographic and etiological characteristics were summarized: mean age 46.55 years, male predominance (68.1%), and female proportion (31.9%). Etiologies were primarily alcohol-induced (approximately 45%), followed by biliary causes (28%), with other factors including hyperlipidemia and drug-related origins. All patients were admitted within 24 h of symptom onset, and baseline clinical features were balanced across groups without significant intergroup disparities.

### 2.2 Inclusion and exclusion criteria

Inclusion Criteria: ➀ Age ≥ 18 years; ➁ Diagnosis of acute pancreatitis per the revised Atlanta classification (2012), defined as typical upper abdominal pain accompanied by serum amylase or lipase levels ≥ 3 times the upper limit of normal; ➂ Hospital admission within 24 h of symptom onset; ➃ Written informed consent obtained from patients or legal guardians for voluntary participation.

Exclusion Criteria: ➀ History of chronic pancreatitis or pancreatic tumors; ➁ Pregnancy or lactation; ➂ Comorbid severe organ diseases, such as end-stage liver disease (Child-Pugh class C), heart failure (New York Heart Association class III-IV), or end-stage renal disease; ➃ Prior pancreatic surgery or interventional therapy before admission; ➄ Incomplete data or loss to follow-up; ➅ Immunodeficiency disorders or long-term immunosuppressant use.

### 2.3 Equipment

Data acquisition and measurements were standardized using multiple medical devices. Abdominal non-contrast and contrast-enhanced computed tomography (CT) scans were performed with a GE Optima CT680 64-Slice CT Scanner. Scanning parameters adhered to standard protocols: tube voltage 120 kV, automatic tube current modulation for optimized radiation dose, slice thickness 5 mm, reconstruction interval 5 mm, contrast agent iohexol (350 mgI/mL) administered at 3 mL/s, with delayed scanning at arterial phase (25–30 s) and portal venous phase (60–70 s). This equipment facilitated calculation of the Modified CT Severity Index (MCTSI) and CT Severity Index (CTSI), with image quality ensured through consistent quality control procedures.

Hematological analysis employed a Sysmex XN-9000 automated hematology analyzer for complete blood counts. Parameters included white blood cell count, lymphocyte count, monocyte count, and neutrophil count, used to compute the lymphocyte-to-monocyte ratio (LMR) and neutrophil-to-lymphocyte ratio (NLR). Sample processing followed manufacturer guidelines, utilizing impedance and fluorescence flow cytometry methods, with daily quality control calibrations to ensure accuracy.

Biochemical analyses were conducted using a Roche Cobas 8000 modular analyzer to measure C-reactive protein (CRP), lactate dehydrogenase (LDH), and urea levels. Methods included immunoturbidimetry for CRP and enzymatic assays for LDH, with original reagents and standardized protocols. Internal quality control complied with Clinical Laboratory Improvement Amendments (CLIA) standards.

Additional equipment included a Philips IntelliVue MX550 monitor for organ failure assessment in the intensive care unit (ICU). This device tracked vital signs such as heart rate, blood pressure, and oxygen saturation, with automated data recording integrated into electronic medical records to maintain objectivity and consistency.

### 2.4 Research methods

(1) Study design: A prospective cohort design was implemented in the Surgery department and gastroenterology department of a single center. Baseline assessments, comprising detailed medical history, physical examination, laboratory tests, and imaging studies, were completed within 24 h of admission. The study adhered to the ethical principles of the Declaration of Helsinki and received approval from the hospital ethics committee.

(2) Data collection: Uniformly trained medical staff recorded data, including demographics (age, sex), etiology (e.g., alcohol-related, biliary), comorbidities (e.g., diabetes, hypertension), and clinical signs. Blood samples were collected at admission using EDTA anticoagulant tubes and processed within 2 h in the central laboratory with the Sysmex XN-9000 analyzer for complete blood counts. LMR was calculated as the absolute lymphocyte count divided by the absolute monocyte count, and NLR as the absolute neutrophil count divided by the absolute lymphocyte count. CT images were independently evaluated by two experienced radiologists blinded to clinical data, with MCTSI and CTSI scores derived. MCTSI scores (range 0–10) assessed pancreatic inflammation, necrosis, and fluid collections, while CTSI scores (range 0–10) evaluated pancreatic and extrapancreatic changes. Discrepancies were resolved by a third radiologist to reach consensus.

(3) Model development: A multivariate logistic regression analysis integrated MCTSI and LMR to develop a novel scoring system (provisionally termed MCTSI-LMR score). Optimization involved determining the optimal LMR cutoff; based on preliminary experiments, LMR < 2.0 was used as a threshold for predicting severe acute pancreatitis, validated via receiver operating characteristic (ROC) curve analysis. Weighted combination employed linear regression, assigning specific weights to MCTSI and LMR, with model fit assessed by likelihood ratio tests. The final score aimed to enable early stratification and prediction.

(4) Follow-up: Patients were followed until discharge or 30 days, whichever occurred first. Follow-up included dynamic monitoring of inflammatory markers (e.g., LMR, NLR) and imaging indices (e.g., MCTSI) at days 1, 3, and 7 post-admission. Outcome data were recorded via electronic medical records and telephone follow-ups to ensure completeness, with oversight by a study coordinator to maintain a dropout rate below 5%.

### 2.5 Observation indicators

(1) Lymphocyte-to-Monocyte Ratio (LMR): Calculated as lymphocyte count divided by monocyte count (dimensionless). Measured via complete blood count, with a normal reference range of 2.0–4.0; decreased LMR correlates with exacerbated inflammatory responses and increased organ failure risk.

(2) Modified CT Severity Index (MCTSI): Scores range from 0 to 10, based on CT assessment of pancreatic inflammation (0–2 points), necrosis (0–4 points), and extrapancreatic complications (0–4 points). Severity categories: 0–2 mild, 3–4 moderate, 5–10 severe; higher scores indicate greater disease severity.

(3) Organ Failure: Evaluated using the modified Marshall scoring system, covering respiratory (oxygenation index), cardiovascular (mean arterial pressure), and renal systems (serum creatinine). Persistent organ failure was defined as a score ≥ 2 lasting ≥ 48 hours, serving as a criterion for severe acute pancreatitis.

(4) Mortality: Recorded as 30-day all-cause mortality, defined as death from any cause between admission and 30 days, verified via hospital records or family confirmation.

(5) ICU Admission Rate: Proportion of cases requiring ICU transfer, reflecting disease severity and resource utilization.

(6) C-Reactive Protein (CRP): Measured by immunoturbidimetry (units: mg/L; normal < 5 mg/L). Elevated levels associate with systemic inflammation and pancreatic necrosis.

(7) Neutrophil-to-Lymphocyte Ratio (NLR): Calculated as neutrophil count divided by lymphocyte count (dimensionless). Normal reference range 1.0–3.0; increased NLR indicates heightened inflammatory states.

(8) Length of Hospital Stay: Total days from admission to discharge, serving as an indirect indicator of prognosis and healthcare resource consumption.

(9) Lactate Dehydrogenase (LDH): Assessed via enzymatic methods (units: U/L; normal range 120–250 U/L). Elevated levels correlate with cellular damage and severe acute pancreatitis.

### 2.6 Statistical methods

Statistical analyses were performed using SPSS software (version 26.0). Continuous variables (e.g., age, LMR) were described as mean ± standard deviation (if normally distributed) or median and interquartile range (if non-normal). Categorical variables (e.g., sex, etiology) were presented as frequencies and percentages. Group comparisons employed independent samples t-tests (for continuous variables) or Mann-Whitney U tests (for non-parametric data), with multi-group analyses using one-way ANOVA or Kruskal-Wallis tests and *post hoc* Bonferroni correction. Predictive model evaluation involved multivariate logistic regression, with acute pancreatitis severity (binary: severe vs. non-severe) as the dependent variable and independent variables including MCTSI and LMR. Odds ratios (OR) and 95% confidence intervals (CI) were computed, and model goodness-of-fit was assessed via Hosmer-Lemeshow tests. Model performance was evaluated through receiver operating characteristic (ROC) curve analysis, calculating area under the curve (AUC), sensitivity, specificity, positive predictive value, and negative predictive value, with comparisons to existing systems such as BISAP and CTSI scores. Survival analysis utilized Kaplan-Meier methods to estimate 30-day mortality, with intergroup differences tested by log-rank tests; multivariate survival analysis employed Cox proportional hazards models, adjusting for potential confounders like age and etiology. All statistical tests were two-sided, with *p* < 0.05 considered significant.

## 3 Results

### 3.1 Comparison of baseline characteristics

**FIGURE 1:**
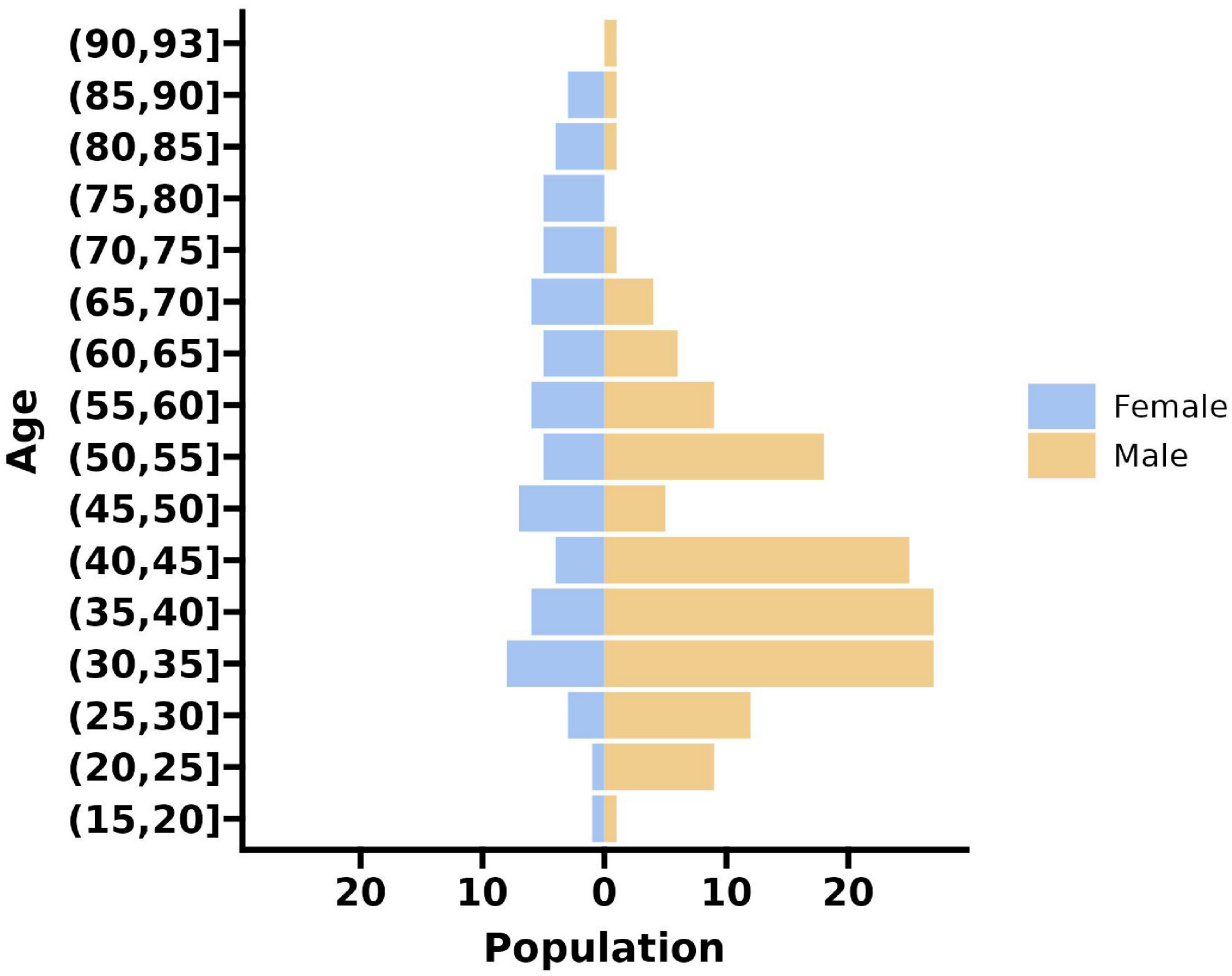
Distribution map of gender and age.

Significant differences were observed among the mild, moderately severe, and severe groups regarding inflammatory markers (CRP, LDH, NLR, LMR) and radiological scores (MCTSI, CTSI), with the severe group demonstrating higher values across all parameters (*P* < 0.05). In contrast, no statistically significant differences were identified in demographic characteristics or specific comorbidities (*P* > 0.05) (see Table 1 and Figure 1).

**TABLE 1:** Comparison of baseline characteristics among the three groups.

Parameter	Mild group (*n* = 65)	Moderately severe group (*n* = 108)	Severe group (*n* = 43)	Statistic (*t*/χ^2^)	*P*-value
Age (years)	44.23 ± 15.67	47.89 ± 16.34	48.91 ± 16.78	*F* = 1.892	0.153
Male/Female (n)	44/21	73/35	30/13	χ^2^ = 0.082	0.960
Alcoholic etiology (n,%)	28 (43.1)	50 (46.3)	20 (46.5)	χ^2^ = 0.194	0.908
Biliary etiology (n,%)	18 (27.7)	31 (28.7)	12 (27.9)	χ^2^ = 0.031	0.985
CRP (mg/L)	35.62 ± 12.34	98.45 ± 35.67	185.73 ± 50.12	*F* = 205.637	<0.001
LDH (U/L)	245.78 ± 85.23	387.45 ± 120.34	598.56 ± 185.67	*F* = 138.294	<0.001
Urea (mmol/L)	5.23 ± 1.45	6.78 ± 2.01	9.45 ± 3.12	*F* = 52.816	<0.001
NLR	3.45 ± 1.23	7.89 ± 2.45	12.34 ± 3.67	*F* = 195.328	<0.001
LMR	3.12 ± 0.89	1.98 ± 0.56	1.23 ± 0.45	*F* = 125.473	<0.001
MCTSI score	2.12 ± 0.78	4.56 ± 1.23	7.89 ± 1.45	*F* = 298.456	<0.001
CTSI score	2.01 ± 0.67	4.23 ± 1.12	7.45 ± 1.67	*F* = 276.891	<0.001
APACHE II on admission	5.67 ± 2.12	10.23 ± 3.45	16.78 ± 4.56	*F* = 159.324	<0.001
BISAP score on admission	0.89 ± 0.34	1.56 ± 0.67	2.78 ± 0.89	*F* = 118.765	<0.001
Diabetes (n,%)	12 (18.5)	25 (23.1)	10 (23.3)	χ^2^ = 0.572	0.751
Hypertension (n,%)	15 (23.1)	30 (27.8)	12 (27.9)	χ^2^ = 0.482	0.786

### 3.2 Comparison of observational indicators

The severe group exhibited the lowest LMR values, while the highest values were observed for MCTSI score, organ failure incidence, mortality, ICU admission rate, CRP, NLR, hospital stay, and LDH. Intergroup comparisons revealed statistically significant differences (*t* = 125.473, 298.456; χ^2^ = 98.765, 45.678, 112.345; *F* = 205.637, 195.328, 136.789, 138.294; *P* < 0.05) (see Table 2).

**TABLE 2:** Comparison of observational indicators among the three groups.

Parameter	Mild group (*n* = 65)	Moderately severe group (*n* = 108)	Severe group (*n* = 43)	Statistic (*t*/χ^2^)	*P*-value
LMR	3.12 ± 0.89	1.98 ± 0.56	1.23 ± 0.45	*F* = 125.473	<0.001
MCTSI score	2.12 ± 0.78	4.56 ± 1.23	7.89 ± 1.45	*F* = 298.456	<0.001
Organ failure (n,%)	0 (0.0)	15 (13.9)	35 (81.4)	χ^2^ = 98.765	<0.001
Mortality (n,%)	0 (0.0)	2 (1.9)	10 (23.3)	χ^2^ = 45.678	<0.001
ICU admission (n,%)	0 (0.0)	20 (18.5)	38 (88.4)	χ^2^ = 112.345	<0.001
CRP (mg/L)	35.62 ± 12.34	98.45 ± 35.67	185.73 ± 50.12	*F* = 205.637	<0.001
NLR	3.45 ± 1.23	7.89 ± 2.45	12.34 ± 3.67	*F* = 195.328	<0.001
Hospital stay (days)	7.23 ± 2.34	14.56 ± 5.67	28.45 ± 10.12	*F* = 136.789	<0.001
LDH (U/L)	245.78 ± 85.23	387.45 ± 120.34	598.56 ± 185.67	*F* = 138.294	<0.001

### 3.3 Multivariate logistic regression analysis

Using acute pancreatitis severity (dichotomized as severe vs. non-severe) as the dependent variable, parameters with *P* < 0.05 in univariate analysis were incorporated into a multivariate logistic regression model. The results indicated that MCTSI score and NLR were positively associated with the risk of severe acute pancreatitis (OR = 1.578 and 1.264, respectively; *P* < 0.05), whereas LMR was negatively correlated (OR = 0.454, *P* < 0.05). CRP did not demonstrate independent predictive significance in the multivariate model (*P* > 0.05) (see Table 3).

**TABLE 3:** Multivariate logistic regression analysis for predicting severe acute pancreatitis.

Factor	B	SE	Wald	*P*-value	OR	95% CI
MCTSI	0.456	0.089	26.234	<0.001	1.578	1.324–1.879
LMR	−0.789	0.123	41.156	<0.001	0.454	0.356–0.578
NLR	0.234	0.067	12.198	0.001	1.264	1.109–1.441
CRP	0.012	0.008	2.25	0.134	1.012	0.996–1.028

### 3.4 Predictive performance of the MCTSI-LMR score

Based on logistic regression results, the MCTSI-LMR score was constructed using the formula: MCTSI × 0.456 − LMR × 0.789 + NLR × 0.234. ROC curve analysis demonstrated that the MCTSI-LMR score predicted severe acute pancreatitis with an AUC of 0.912, sensitivity of 86.7%, and specificity of 88.9%. The AUC of the MCTSI-LMR score was significantly higher than those of MCTSI, LMR, NLR, and BISAP scores (*Z* = 3.456, 4.123, 4.567, 5.678; *P* < 0.05), indicating superior predictive performance (see Table 4).

**TABLE 4:** ROC curve analysis of various scores for predicting severe acute pancreatitis.

Score	AUC	95% CI	Sensitivity (%)	Specificity (%)	*P*-value
MCTSI-LMR	0.912	0.867–0.945	86.7	88.9	<0.001
MCTSI	0.845	0.789–0.891	79.1	82.3	<0.001
LMR	0.801	0.745–0.856	74.4	78.9	<0.001
NLR	0.783	0.723–0.834	72.1	76.5	<0.001
BISAP	0.756	0.698–0.812	69.8	73.2	<0.001

### 3.5 Dynamic changes in monitoring indicators

**FIGURE 2:**
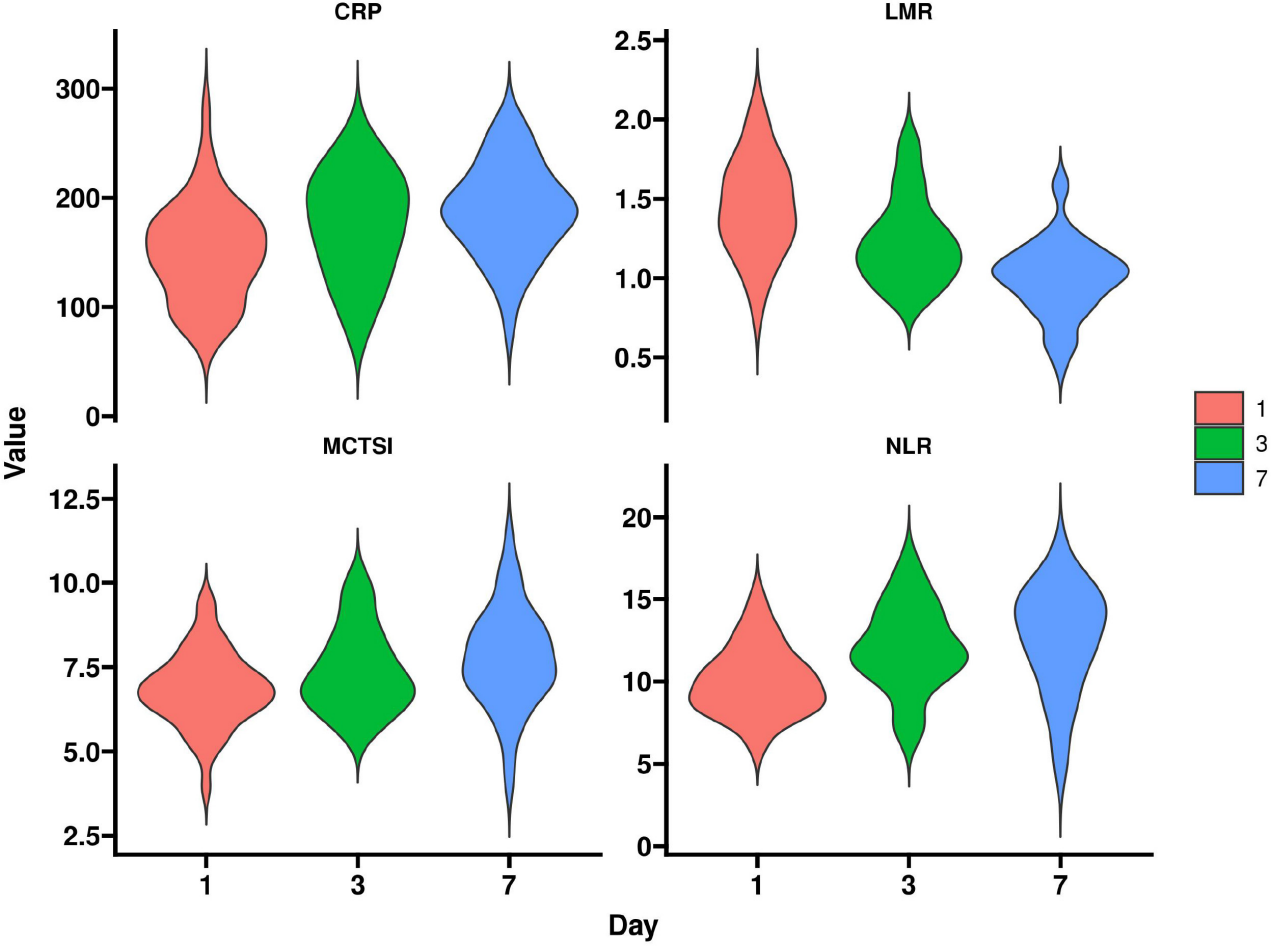
Dynamic changes in indicators in the severe group.

In the severe group, LMR decreased over time after admission, while MCTSI, NLR, and CRP increased, with dynamic trends consistent with disease progression. Comparisons of indicators on days 1, 3, and 7 were statistically significant (*P* < 0.05). Specific data are presented in Table 5 and Figure 2.

**TABLE 5:** Dynamic changes in indicators in the severe group (*n* = 43).

Parameter	Day 1	Day 3	Day 7	Statistic (F)	*P*-value
LMR	1.45 ± 0.34	1.23 ± 0.29	1.01 ± 0.23	*F* = 15.678	< 0.001
MCTSI	6.78 ± 1.23	7.45 ± 1.34	7.89 ± 1.45	*F* = 8.912	0.001
NLR	10.12 ± 2.34	11.45 ± 2.67	12.34 ± 3.01	*F* = 7.345	0.002
CRP (mg/L)	150.45 ± 40.12	170.89 ± 45.67	185.73 ± 50.12	*F* = 6.789	0.003

### 3.6 Cox regression analysis for predicting 30-day mortality

Kaplan-Meier survival curves revealed that the severe group had significantly lower 30-day survival compared to the mild and moderately severe groups (log-rank χ^2^ = 45.678, *P* < 0.001). Cox regression analysis indicated that the MCTSI-LMR score was an independent predictor of survival (HR = 1.456, 95% CI 1.234–1.718, *P* < 0.001). Specific data are shown in Table 6.

**TABLE 6:** Cox regression analysis for predicting 30-day mortality.

Factor	B	SE	Wald	*P*-value	OR	95% CI
MCTSI-LMR score	0.376	0.089	17.832	<0.001	1.456	1.234–1.718
Age	0.012	0.008	2.250	0.134	1.012	0.996–1.028
Organ failure	0.567	0.123	21.234	<0.001	1.763	1.385–2.245

## 4 Discussion

In baseline comparisons, no significant differences were observed among the three patient groups (mild, moderately severe, and severe) regarding demographic features such as age, sex, or etiology distribution. However, marked disparities were noted in inflammatory markers (e.g., C-reactive protein, lactate dehydrogenase) and imaging scores (e.g., MCTSI, CTSI). This suggests that acute pancreatitis severity is more closely associated with immunological and morphological changes rather than traditional demographic factors (12, 13). Inflammatory markers like C-reactive protein and lactate dehydrogenase reflect systemic inflammatory responses and cellular injury, while imaging scores directly quantify local pancreatic damage. Early activation of the innate immune system in acute pancreatitis triggers cytokine release and oxidative stress, exacerbating tissue injury and organ dysfunction—a dynamic process more pronounced in severe cases, explaining the observed baseline differences (14, 15).

Regarding observational indicators, the severe acute pancreatitis group exhibited significantly lower LMR values alongside elevated MCTSI scores, organ failure rates, and other inflammatory markers. This implies that LMR, as an easily accessible hematologic marker, may inversely correlate with disease severity and aid in identifying high-risk patients (16, 17). Mechanistically, LMR serves as an immune balance indicator; its decline often signifies lymphocyte depletion and monocyte activation, aligning with hyperinflammation and immunosuppression in acute pancreatitis. Our findings suggest that LMR modulates the interplay between T lymphocytes and monocytes, influencing the release of cytokines such as interleukins and tumor necrosis factor, thereby exacerbating microcirculatory impairment and apoptosis, ultimately driving organ failure and adverse outcomes (18).

Multivariate logistic regression identified MCTSI, LMR, and the Neutrophil-to-Lymphocyte Ratio (NLR) as independent predictors of severe acute pancreatitis, whereas C-reactive protein lacked independent significance. This highlights the stability of a multi-parameter predictive model over single-variable approaches. MCTSI quantifies structural pancreatic changes, while LMR and NLR reflect systemic inflammation, collectively capturing the multidimensional nature of the disease. MCTSI directly assesses local pathology through necrosis and inflammation extent, whereas LMR and NLR influence systemic inflammatory cascades via immune cell ratios and cytokine networks, synergistically promoting disease progression—consistent with the established immunoinflammatory axis theory (19, 20).

In predictive performance evaluation, the MCTSI-LMR score demonstrated a higher area under the curve, with superior sensitivity and specificity compared to MCTSI, LMR, or NLR alone. This supports its clinical utility as a composite tool for early stratification (21). By integrating imaging severity and immune-inflammatory status, the MCTSI-LMR score comprehensively addresses the multifactorial nature of acute pancreatitis. Its weighted combination of MCTSI’s structural data and LMR’s immunological profile enhances accuracy in predicting pancreatic necrosis and systemic complications, aligning with personalized medicine principles (22).

Dynamic monitoring revealed progressive LMR decline alongside rising MCTSI, NLR, and C-reactive protein in severe cases, indicating sustained immune dysregulation and worsening inflammation over time. This underscores the value of serial assessments for timely therapeutic adjustments (23). The LMR trajectory likely mirrors lymphocyte depletion and monocyte expansion, driven by a vicious cycle of immunosuppression and inflammatory amplification. Persistent pancreatic injury exacerbates oxidative stress and cytokine storms, depleting antioxidant reserves and activating innate immunity, thereby aggravating microvascular leakage and multi-organ dysfunction—a process emphasizing the need for early intervention (24).

Survival analysis confirmed the MCTSI-LMR score as an independent predictor of 30-day mortality, unaffected by age or other factors. This positions the score as both a stratification and prognostic tool for clinical decision-making (25). By synthesizing disease severity and immune status, the score directly correlates with survival outcomes. It reflects the interplay between structural damage and systemic inflammation, influencing apoptosis and organ failure pathways. High scores may indicate severe immune paralysis and impaired tissue repair, resonating with prior survival models (26). Beyond its prognostic value, the identification of a low LMR at admission may have potential therapeutic implications. It could serve as an early, objective indicator to trigger intensified monitoring and aggressive supportive care, aligning with the current management paradigm of early goal-directed therapy in severe AP. Moreover, such a biomarker might be valuable for patient stratification in future clinical trials exploring immunomodulatory strategies aimed at mitigating the dysregulated immune response characteristic of severe disease. While direct therapeutic modulation of the LMR (e.g., via immunotherapy to stimulate lymphopoiesis) is not a current standard of care, its association with outcomes underscores its role as a marker of the underlying pathological immune state, guiding overall clinical vigilance and resource allocation.

### 4.1 Safety issues

Subjects did not report any side effects.

### 4.2 Study limitations

Limitations include the single-center design and modest sample size, which may limit generalizability and statistical power. Future multicenter prospective validation across diverse populations is warranted to assess the score’s robustness. Additionally, exploring its utility in treatment response prediction and long-term follow-up could further refine acute pancreatitis management. It is important to acknowledge a limitation regarding the MCTSI assessment timing. As pancreatic necrosis often requires 48–72 h to become fully demarcated on CT, evaluation within the first 24 h, as performed in our study, may underestimate the true extent of necrotic tissue and thus the final MCTSI score. This is an inherent constraint of pursuing very early stratification. However, the primary objective of our study was to develop a tool applicable at initial patient presentation. Despite this potential for underestimation, MCTSI at 24 h remained a strong independent predictor of severity in our cohort. The integration with LMR, a dynamic hematological marker, may partially compensate for this early radiological limitation by providing concurrent systemic inflammatory information.

## 5 Conclusion

The MCTSI-LMR score demonstrates superior predictive performance in early stratification of acute pancreatitis. Its integration of imaging and inflammatory biomarkers offers a practical clinical tool, potentially enhancing risk assessment and personalized therapy, thereby advancing precision medicine in acute pancreatitis care.

## Data Availability

The original contributions presented in this study are included in this article/supplementary material, further inquiries can be directed to the corresponding author.
